# Identification and characterization of methicillin-resistant *Staphylococcus* spp. isolated from surfaces near patients in an intensive care unit of a hospital in southeastern Brazil

**DOI:** 10.1590/0037-8682-0244-2020

**Published:** 2020-11-06

**Authors:** Luciano Freitas Fernandes, Geziella Áurea Aparecida Damasceno Souza, Anna Christina de Ameida, Léia Cardoso, Mauro Aparecido de Sousa Xavier, Talles Patrick Prates Pinheiro, Guilherme Henrique Santos da Cruz, Hellen Fonseca Silva Dourado, Wender Soares Silva, Alessandra Rejane Ericsson de Oliveira Xavier

**Affiliations:** 1 Universidade Estadual de Montes Claros, Departamento de Clínica Médica, Montes Claros, MG, Brasil.; 2 Hospital Santa Casa de Montes Claros, Comitê de Controle de Infecções Relacionadas à Assistência à Saúde, Montes Claros, MG, Brasil.; 3 Universidade Federal de Minas Gerais, Laboratório de Sanidade Animal, Montes Claros, MG, Brasil.; 4 Universidade Estadual de Montes Claros, Departamento de Fisiopatologia, Montes Claros, MG, Brasil.; 5 Hospital Santa Casa de Montes Claros, Laboratório de Microbiologia, Montes Claros, MG, Brasil.

**Keywords:** Coagulase-negative *Staphylococcus*, Equipment contamination, Methicillin resistance

## Abstract

**INTRODUCTION:**

Contaminated hospital environments contribute to the transmission of microorganisms associated with healthcare. Contaminated surfaces handled by patients or healthcare professionals are a source of microorganism transmission by hand. Methicillin-resistant *Staphylococcus* bacteria are among the main agents responsible for increasing healthcare-associated infections in Brazil and worldwide.

**METHODS:**

The objective of this study was to screen and characterize methicillin-resistant *Staphylococcus* spp. on surfaces near patients in an intensive care unit. Microbiological samples, collected from ten beds in an intensive care unit with five sampling sites, were inoculated into a methicillin-resistant *Staphylococcus aureus* chromogenic medium. MALDI-TOF and PCR analyses were used to identify the bacteria. Antimicrobial susceptibility was determined using the disk diffusion test. The presence of the *mecA* gene was investigated using PCR.

**RESULTS:**

We observed that 44 out of the 50 sampling sites presented grown isolates in the methicillin-resistant *Staphylococcus aureus* medium. The incidence of isolated microorganisms on the right side rail, left side rail, tables, infusion pump keypad, and cardiac monitor were 18.8 %, 36.7 %, 10.9 %, 2.4 %, and 31 %, respectively. The 42 isolates included in this study were identified as coagulase-negative *Staphylococcus*. All of these microorganisms were multidrug-resistant and *mecA* gene-positive.

**CONCLUSIONS:**

This study identified the presence of methicillin-resistant coagulase-negative *Staphylococcus* on the beds of an intensive care unit, providing evidence for the necessity of assertive actions to decrease the risk of healthcare-associated infections at the site.

## INTRODUCTION

Healthcare-associated infections (HAIs) are infections that patients acquire while receiving healthcare^1^. HAIs include cross-infection among patients infected or colonized with pathogenic bacteria, which can be transmitted from patient to patient directly or indirectly through vomit, devices, healthcare professionals, companions, and the environment around the patients^2^. HAIs have an impact on morbidity, mortality, and length of patient stay in nosocomial units, leading to increased mean costs of hospitalization^3^. The environment surrounding the patients is an important source of HAI microorganisms. Surfaces such as side rails, medical equipment, support tables, and curtains, among others, can become reservoirs of harmful microorganisms that can be transmitted to patients either directly, when the patient comes into contact with the medium, or indirectly, by contamination of the hands and gloves of health professionals^4^. Surface cleaning and disinfection are extremely important to reduce the occurrence of microbial contamination and consequently the risk of HAIs. This cleaning process is highly complex and multidimensional, involving the physical friction of the surfaces to remove organic and inorganic materials, and then using a disinfectant solution, as well as monitoring strategies to ensure proper hygiene procedures^5^ ^,^ ^6^.

Bacteria of the genus *Staphylococcus* are the main cause of HAIs; their high prevalence on the skin of patients facilitates infection after a medical procedure breaks the barrier, or may be associated with decreased immunity. Coagulase-negative *Staphylococcus* (CoNS) strains are the main etiological agents of a series of infectious processes acquired in a hospital environment. In 2018, CoNS were the main causative agents of catheter-associated bloodstream infection in ICUs in Brazil^7^. The occurrence of these CoNS-related HAIs has several predisposing factors, including the immune and health status of the patient and the environment close to the patient, which can be a source of both origin and transmission^8^ ^,^ ^9^. Another characteristic that makes CoNS-related HAIs worrisome is their increased resistance to beta-lactam antibiotics, especially methicillin^10^.

Resistance mechanisms against this class of antibiotics, developed by bacteria of the genus *Staphylococcus* spp., include two specificities that are important for their prevalence: the production of the beta-lactamase enzyme encoded by the *blaZ* gene, and the modified effector mechanism of beta-lactam antibiotics with the modification of the PBP binding protein into PBP2A protein, which has a low affinity for penicillin-binding, encoded by the *mecA* gene and its homologs, including *mecALGA251* ^10^. As the main human skin colonizer with a high survival capacity in inert, inhospitable environments with low substrate levels, CoNS often colonize the environment near the patients. They can be differentiated from *S. aureus* at the species level through PCR identification of the *femA* gene (an essential factor for methicillin resistance). This gene encodes a 48-50 kDa protein recognized as a specific factor and is contained in the chromosomes of these pathogens^11^. Thus, the objective of this study was to screen and characterize methicillin-resistant *Staphylococcus* spp. on surfaces near patients in the ICU of a hospital in southeastern Brazil.

## METHODS

### Microorganism sampling and collection sites

The samples included in this study were composed of microorganisms isolated from hospital ICU bed surfaces, not involving patients (or their data) or any approach to health professionals. The sample collection was authorized by the hospital where the research was conducted.

**FIGURE 1:**
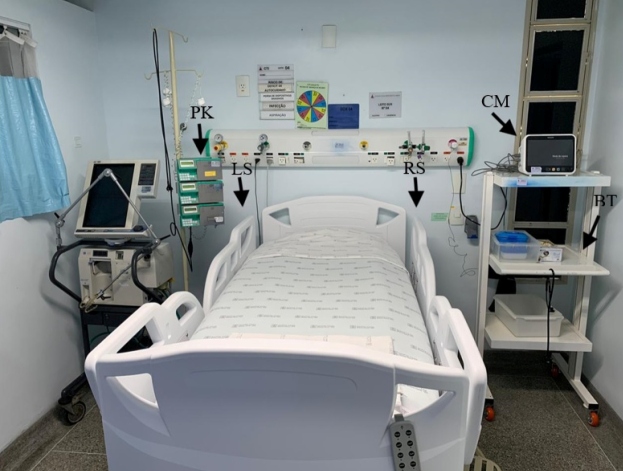
Sampling sites in an ICU. The arrows indicate the sampling locations: infusion pump keypad (PK), left side rail (LS), right side rail (RS), cardiac monitor (CM) and bedside table (BT).

The study was conducted in a general tertiary-level hospital located in the southeast region of Brazil. It has 397 beds and 3 ICUs, of which the general ICU, containing 10 beds, was chosen for sample collection. The rationale for choosing the general ICU was based on the fact that this environment receives patients from different hospital sectors and with different clinical conditions, increasing the variability of their microbiota, with a greater chance of analyzing the real epidemiological profile of the institution. Each of the 10 ICU beds included 5 sampling sites: right side rail (RS), left side rail (LS), bedside table (BT), infusion pump keypad (PK), and cardiac monitor (CM) (Figure 1). These points were chosen based on the frequency with which they are manipulated/touched, acting as potential contamination sources for healthcare professionals and patients^12^ ^,^ ^13^. Surface sampling was performed around ICU bed surfaces before any cleaning procedure was performed. The sampling procedure was performed with patients inside the room. The samples were collected on October 24, 2019, using swabs immersed in saline solution for sampling a 5 cm² area. Subsequently, the swabs were depleted on Petri dishes containing a methicillin-resistant *Staphylococcus aureus* (MRSA) chromogenic growth medium (Probac do Brasil, Brazil). The plates were incubated at 37°C for 24 h. After the incubation period, colony-forming units (CFUs) were counted and registered on a form.

### Microorganism identification

 Sampling sites where no growth could be observed were excluded from the study. A total of 42 colonies from different sampling sites were analyzed using the Matrix-Assisted Laser Desorption Ionization-Time of Flight Mass Spectrometry (MALDI-TOF MS) proteomic identification approach in the AQUACEN laboratory of the Veterinary School of the Federal University of Minas Gerais (UFMG), according to the methodology previously described by Assis et al.^14^, using a Microflex TM MALDI-TOF MS device (Bruker Daltonics, USA). Furthermore, the genomic PCR method was used to identify *S. aureus* at the species level through the detection of the *femA* marker gene, as described by Xavier et al.^11^

### Genomic analyses

The bacteria were selected on plates containing MRSA medium, transferred to Brain Heart Infusion (BHI) broth (Laborclin, Brazil), inoculated onto mannitol salt agar (Laborclin, Brazil) plates, and incubated at 37°C for 24 h. The growing colonies were analyzed using the DNA extraction procedure described by Gu et al.^15^ The integrity and quantification of the extracted DNA were verified by 1.0 % (w/v) agarose gel electrophoresis. This material was used for PCR . All primers used in this study were synthesized by GenOne Biotechnologies, Brazil.

PCR tests were used for *femA* gene detection, a species-specific marker for *S. aureus,* and *mecA* gene detection, a specific marker for methicillin resistance, as described by Xavier et al.^17^The reactions were performed using a mix containing 2× Go Taq Green Master Mix® (Promega Corporation, USA), 10 µM of each primer, and 50 ng of DNA, with a final reaction volume of 50 µL. PCR was performed in a Veriti Thermal Cycler (Applied Biosystems,USA). The PCR thermal conditions were described by Xavier et al.^17^ The amplicons were visualized on 1.5 % (w/v) agarose gel stained with ethidium bromide and photo-documented. *S. aureus* ATCC 43300, *S. aureus* ATCC 29213, and *S. aureus* 25923 strains were used as controls for PCR.

### Antimicrobial sensitivity profile

The susceptibility to beta-lactam antibiotics was determined by the disk diffusion test according to the Clinical & Laboratory Standards Institute (CLSI) guidelines^16^, using the following antimicrobials (Laborclin): oxacillin, 1 μg (OXA); erythromycin, 15 μg (ERI); cefoxitin, 30 μg (CFO); cefazolin, 30 μg (CFZ), cefuroxime, 30 μg (CRX); ciprofloxacin, 5 μg (CIP); sulfatrim, 25 μg (SUT); amikacin, 30 μg (AMI); clindamycin, 2 μg (CLI); cephalothin, 30 μg (CFL); and gentamicin, 10 μg (GEN).

## RESULTS

This study demonstrated environmental surface contamination through the collection of microbiological samples from 50 surfaces near patients in an ICU. We observed that 44 out of the 50 samples grew on MRSA medium-containing plates. Although they grew on MRSA medium, two samples did not grow in mannitol salt agar and were therefore excluded.

**FIGURE 2:**
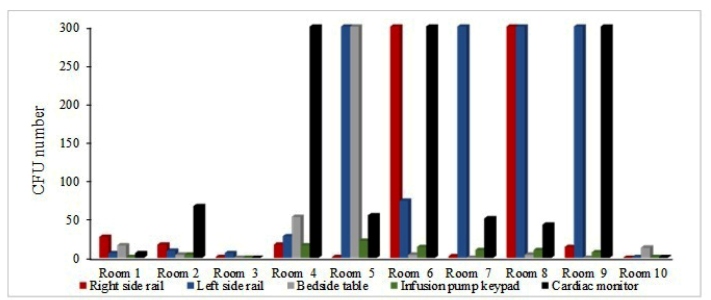
Result of the total viable count of microorganisms sampled on different surfaces in the intensive care unit rooms.

Beds 4, 5, 6, 7, 8, and 9 presented contamination levels higher than 300 CFUs (Figure 2). The overall incidence of microorganisms in samples collected from RS, LS, BT, PK, and CM were 18.8 %, 36.7 %, 10.9 %, 2.4 %, and 31 %, respectively. This result revealed the dissemination of phenotypically methicillin-resistant strains on the ten sampling beds.

Proteomic genus identification confirmed that all isolates were *Staphylococcus* bacteria; however, species identification was only possible for 45.22 % (19/42) of the isolates. The species isolated from the surface samples of this study were all CoNS. The microorganisms identified were *S. haemolyticus* (11), *S. epidermidis* (4), *S. capitis* (2), and *S. cohnii* (1) (Table 1).

**TABLE 1:** Identification and characterization of the phenotypic antimicrobial resistance profile of microorganisms isolated from bed surfaces of an Intensive Care Unit in a hospital in Southeastern Brazil.

Nº	Location site	Gene	Gene	Proteomic identification	Phenotypic antimicrobial resistance profile
		femA	mecA	MALDI-TOF	
1	Right side rail - Bed 1	-	+	*Staphylococcus* spp	OXA; ERI; CFO; CRX; SUT; AMI; CLI; GEN
2	Left side rail - Bed 1	-	+	*Staphylococcus* spp	OXA; ERI; CFO; CRX; SUT; AMI; CLI; GEN
3	Bedside table - Bed 1	-	+	*Staphylococcus* spp	OXA; ERI; CFO; CIP; CRX; AMI; CLI; GEN
4	Pump keypad - Bed 1	-	+	*Staphylococcus* spp	OXA; ERI; CFO; CFZ; CRX; CLI; GEN
5	Cardiac monitor - Bed 1	-	+	*Staphylococcus hominis*	OXA; ERI; CFO; CFZ; CIP; SUT; CRX; CLI
6	Right side rail - Bed 2	-	-	*Staphylococcus haemolyticus*	OXA; CFO; CFZ; CRX; SUT; AMI; CLI
7	Left side rail - Bed 2	-	+	*Staphylococcus* spp	OXA; ERI; CFO; SUT; AMI; CLI; GEN
8	Bedside table - Bed 2	-	+	*Staphylococcus* spp	OXA; ERI; CFO; CFZ; CRX; CLI; GEN.
9	Pump keypad - Bed 2	-	+	*Staphylococcus* spp	OXA; ERI; CFO; CRX; SUT; AMI; CLI; GEN
10	Cardiac monitor - Bed 2	-	+	*Staphylococcus* spp	OXA; ERI; CFO; CRX; SUT; AMI; CLI; GEN
11	Right side rail - Bed 3	-	-	*Staphylococcus* spp	OXA; ERI; CFO; CFZ; CRX; CIP; SUT; CLI; CFL; GEN
12	Left side rail - Bed 3	-	-	*Staphylococcus haemolyticus*	OXA; ERI; CFO; CFZ; CRX; CIP; SUT; CLI; CFL; GEN
13	Right side rail - Bed 4	-	+	*Staphylococcus epidermidis*	OXA; ERI; CFO; CRX; CIP; AMI; CLI; GEN
14	Left side rail - Bed 4	-	+	*Staphylococcus* spp	OXA; ERI; CFO; CFZ; CRX; CIP; SUT; AMI; CLI; GEN
15	Bedside table - Bed 4	-	+	*Staphylococcus haemolyticus*	OXA; ERI; CFO; CFZ; CRX; CIP; SUT; CLI; CFL; GEN
16	Pump keypad - Bed 4	-	+	*Staphilococcus epidermidis*	OXA; ERI; CFO; CFZ; CRX; CIP; SUT; AMI; CLI
17	Cardiac monitor - Bed 4	-	+	*Staphylococcus* spp	OXA; ERI; CFO; CFZ; CRX; CIP; SUT; CLI; CFL; GEN
18	Right side rail - Bed 5	-	+	*Staphylococcus* spp	OXA; ERI; CFO; CRX; CIP; AMI; CLI;
19	Left side rail - Bed 5	-	+	*Staphylococcus captis*	OXA; ERI; CFO; CFZ; CRX; SUT; AMI; CLI; GEN
20	Bed side table - Bed 5	-	+	*Staphylococcus* spp	OXA; ERI; CFO; AMI; CLI; GEN
21	Pump keypad - Bed 5	-	+	*Staphylococcus haemolyticus*	OXA; ERI; CFO; CFZ; CRX; CIP; SUT; CLI; CFL; GEN
22	Cardiac monitor - Bed 5	-	-	*Staphylococcus haemolyticus*	OXA; ERI; CFO; CFZ; CRX; CIP; SUT; CLI; CFL; GEN
23	Right side rail - Bed 6	-	+	*Staphylococcus* spp	OXA; ERI; CFO; CRX; SUT; AMI; CLI; GEN
24	Left side rail - Bed 6	-	+	*Staphylococcus* spp	OXA; ERI; CFO; CFZ; CRX; CIP; AMI; CLI; CFL; GEN
25	Bedside table - Bed 6	-	+	*Staphylococcus* spp	OXA; ERI; CFO; CFZ; CIP; SUT; AMI; CLI; CFL; GEN
26	Pump keypad - Bed 6	-	+	*Staphylococcus haemolyticus*	OXA; ERI; CFO; CFZ; CRX; CIP; AMI; CLI; CFL; GEN
27	Cardiac monitor - Bed 6	-	+	*Staphylococcus epidermidis*	OXA; ERI; CFO; CRX; CIP; SUT; AMI; CLI; GEN
28	Right side rail - Bed 7	-	+	*Staphylococcus* spp	OXA; ERI; CFO; CFZ; CRX; CIP;
29	Left side rail - Bed 7	-	+	*Staphylococcus haemolyticus*	OXA; ERI; CFO; CRX; SUT; AMI; CLI; GEN
30	Pump keypad - Bed 7	-	+	*Staphylococcus haemolyticus*	OXA; ERI; CFO; CFZ; CRX; CIP; SUT; CLI; CFL; GEN
31	Cardiac monitor - Bed 7	-	+	*Staphylococcus* spp	OXA; ERI; CFO; CRX; SUT; AMI; CLI; GEN
32	Right side rail - Bed 8	-	+	*Staphylococcus* spp	OXA; ERI; CFO; CRX; SUT; AMI; CLI; GEN
33	Left side rail - Bed 8	-	+	*Staphylococcus captis*	OXA; ERI; CFO; CFZ; CRX; CIP; SUT; AMI; CLI; GEN
34	Bedside table - Bed 8	-	+	*Staphylococcus* spp	OXA; ERI; CFO; CFZ; CRX; CIP; SUT; CLI; GEN
35	Cardiac monitor - Bed 8	-	+	*Staphylococcus* spp	OXA; ERI; CFO; CRX; SUT; AMI; CLI; GEN
36	Right side rail - Bed 9	-	+	*Staphylococcus haemolyticus*	OXA; ERI; CFO; CFZ; CRX; CIP; SUT; CLI; CFL; GEN
37	Left side rail - Bed 9	-	+	*Staphylococcus haemolyticus*	OXA; ERI; CFO; CFZ; CRX; CIP; SUT; CLI; CFL; GEN
38	Pump keypad - Bed 9	-	+	*Staphylococcus cohnii*	OXA; ERI; CFO; CFZ; CRX; SUT; CLI; CFL;
39	Cardiac monitor - Bed 9	-	+	*Staphylococcus haemolyticus*	OXA; ERI; CFO; CFZ; CRX; CIP; SUT; CLI; CFL; GEN
40	Left side rail - Bed 10	-	+	*Staphylococcus* spp	OXA; ERI; CFO; CFZ; CRX; SUT; AMI; CLI; CFL; GEN
41	Bedside table - Bed 10	-	+	*Staphylococcus* spp	OXA; ERI; CFO; SUT; CLI; GEN
42	Cardiac monitor - Bed 10	-	+	*Staphylococcus epidermidis*	OXA; ERI; CFO; CRX; CIP; AMI; CLI; GEN

**OXA:** Oxacillin; **ERI:** Erythromycin; **CFO:** Cefoxitin; **CFZ:** Cefazolin; **CRX:** cefuroxime; **CIP**: Ciprofloxacin; **SUT:** sulfatrim; **AMI:** amikacin; **CLI:** clindamycin; **CFL:** cephalothin; **GEN:** gentamicin.

**FIGURE 3:**
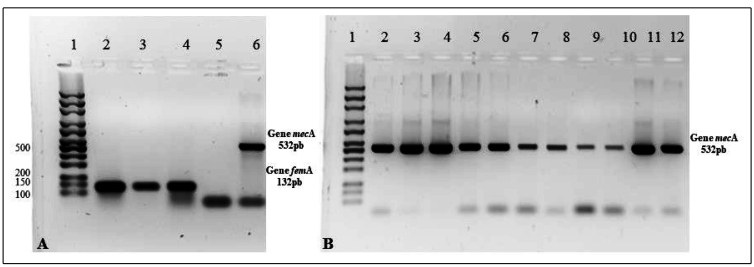
Result of PCR analysis to detect *femA* and *mecA* genes among *Staphylococcus* isolated from different surfaces of an ICU bed. Panel A. Standardization of the PCR for detection of *femA* and *mecA* genes in standard strains of *Staphylococcus aureus*. Line 1: Mid-Range DNA Ladder molecular weight marker (Cellco Biotechnology); Lines 2 to 4: Amplification of the *femA* gene in standard strains of *S.aureus* ATCC 43300, *S. aureus* ATCC 29230, and *S. aureus* 25923. Lines 5 and 6: negative and positive controls, respectively, for the PCR of the *mecA* gene in strains of *S. aureus* ATCC 29230 and *S.aureus* ATCC 43300. Panel B. Result of PCR analysis for detection of the *mecA* gene among the isolated *Staphylococcus*. Line 1: Mid-Range DNA Ladder molecular weight marker (Cellco Biotechnology); Lines 2 to 12: Amplification of the *mecA* gene in isolates 32 to 42.

Due to the low species identification index obtained by MALDI-TOF, PCR was used to trace the *femA* gene, a species-specific marker for *S. aureus*, in all isolates. Control *S. aureus* strains were used to standardize the PCR for positive *femA* gene identification (Figure 3A). None of the isolates were *femA* gene-positive (Table 1), confirming the species identified using the MALDI-TOF and excluding the possibility that the strains could be identified as *S. aureus.*

Although the isolated strains were not *S. aureus*, they all grew on MRSA medium-containing plates, and their resistance to methicillin was tested at the genomic level by PCR screening for the *mecA* gene (Figure 2). Control *S. aureus* strains were used to standardize the PCR to screen for *mecA* gene-positive samples (Figure 3A). Among all the isolates, 90.47 % (38/42) were *mecA* gene-positive, confirming the phenotypic growth result in MRSA medium, even if they were not *S. aureus* strains*.* Figure 3B shows the *mecA* gene PCR results of representative isolates.

The antimicrobial susceptibility test showed the presence of extensively drug-resistant strains (Table 1). Among the 42 isolates, 100 % (42/42) were resistant to oxacillin, 98 % (41/42) to erythromycin, 100 % (42/42) to cefoxitin, 60 % (25/42) to cefazolin, 81 % (34/42) to ceftriaxone, 50 % (21/42) to ciprofloxacin, 74 % (31/42) to trimethoprim-sulfamethoxazole, 57 % (24/42) to amikacin, 98 % (41/42) to clindamycin, and 86 % (36/42) to gentamicin (Table 1). After the microorganisms were separated by species as identified by MALDI-TOF, the resistance to the tested antibiotics was *Staphylococcus* spp. (microorganisms classified only by genus), 72 %; *Staphylococcus haemolyticus,* 87 %; *Staphylococcus epidermidis*, 73 %; *Staphylococcus capitis*, 82 %; *Staphylococcus hominis*, 64 %; and *Staphylococcus cohnii*, 73 % (Table 2).

**TABLE 2:** Antimicrobial resistance profile tested for each species identified by MALDI-TOF.

	Quantity	OXA	ERI	CFO	CFZ	CRX	CIP	SUT	AMI	CLI	CFL	GEN	Total Resistant
*Staphylococcus spp*	23 (55%)	23	23	23	10	17	7	16	15	22	5	21	182
*Staphylococcus haemolyticus*	11 (26.2%)	11	10	11	10	11	9	10	3	11	9	10	105
*Staphylococcus epidermidis*	4 (9.5%)	4	4	4	1	3	3	2	4	4	0	3	32
*Staphylococcus captis*	2 (4.8%)	2	2	2	2	2	1	1	2	2	0	2	18
*Staphylococcus hominis*	1 (2.4%)	1	1	1	1	0	1	1	0	1	0	0	7
*Staphylococcus cohnii*	1 (2.4%)	1	1	1	1	1	0	1	0	1	1	0	8
Total resistant		42	41	42	25	34	21	31	24	41	15	36	352

OXA: Oxacillin; ERI: Erythromycin; CFO: Cefoxitin; CFZ: Cefazolin; CRX: cefuroxime; CIP: Ciprofloxacin; SUT: sulfatrim; AMI: amikacin; CLI: clindamycin; CFL: cephalothin; GEN: gentamicin.

## DISCUSSION

The presence of bacterial contamination on inanimate surfaces in a hospital environment has attracted the interest of healthcare professionals in order to relate these microorganisms to HAIs. Gram-positive bacteria have been researched as they are more prevalent in these environments, especially in ICUs. Most of the studies on this subject show environmental contamination by *Staphylococcus aureus* ^17^.

CoNS represent a group of microorganisms that are the main definitive or transitory colonizers of human skin and mucous membranes. However, as they live in balance with the human microbiota, for some time the CoNS were considered simple contaminants of biological samples and often underestimated, unlike *S. aureus* ^17^. CoNS are considered pathogens of great relevance to hospital environments, both for their ability to cause infections and to develop antibiotic resistance. They have been considered especially important in ICUs due to their high transmissibility by the hands of healthcare professionals or by indirect contact with contaminated equipment and surfaces^16^ ^,^ ^18^. Species relevant to humans include *S. epidermidis, S. capitis, S. warneri, S. haemolyticus, S. hominis, S. saccharolyticus, S. caprae, S. pasteuri, S. saprophyticus, S. xylosus, S. cohnii, S. simulans, S. auricularis, S. lugdunensis,* and *S. schleiferi.* These microorganisms are considered opportunistic agents, and *S. epidermidis* is the most associated with HAI^19^ ^,^ ^20^.

This study analyzed 50 samples collected in the ICU of a general hospital. Each of the 10 beds had 5 collection sites chosen according to the frequency they are handled by the multidisciplinary team; therefore, they are considered potential sources of contamination.

Of the 50 samples collected, 42 (84 %) showed bacterial growth due to the presence of microorganisms that commonly colonize hands, which spread CoNS on the equipment and surfaces near the patient. In this work, the sampled surfaces were not subjected to any disinfection process prior to sampling collection, since the objective was to track the presence or absence of *Staphylococcus* bacteria. Of the equipment and surfaces analyzed, LS and CM were the most contaminated. A study by Moraes et al. also reported high contamination rates in infusion pumps^21^ and, as in our work, it was not possible to determine the contamination source. Microorganisms could have been carried to the LS and RS by the patients or healthcare professionals’ handling. Studies concerning microorganism phylogenetic approaches could be useful in elucidating the origin of contamination sources. For this purpose, patients’ and healthcare professional’s hands sampling should be investigated and their results should be compared with those of hospital ICU surfaces analyses.

MALDI-TOF MS analysis was performed in a second step for genus identification. Of the samples, 55 % (23/42) were identified as *Staphylococcus* spp. and the other 45 % (19/42) were identified as: 26.2 % (11/42) *Staphylococcus haemolyticus, 9.5 % (*4/42) *Staphylococcus epidermidis,* 4,8 % (2/42) *Staphylococcus capitis,* 2.4 % (1/42) *Staphylococcus hominis,* and 2.4% (1/42) *Staphylococcus cohnii*. The results showed the diversity of CoNS species and similar results were obtained in a study by Bernardi and Pizzolitto, in which the main microorganisms identified were *S. epidermidis* and *S. haemolyticus,* different only by the fact that *S. epidermidis* had the highest prevalence in this study^19^.

PCR analysis of the *femA* gene showed no presence of *Staphylococcus aureus* in any of the samples, confirming the above result, which did not identify the presence of *S. aureus.* The *mecA* gene was present in 95 % (40/42) of the samples. It was absent in only 3 *S. haemolyticus* samples and 1 *S. epidermis* sample*.* Oxacillin resistance without the *mecA* gene may occur for other reasons, such as beta-lactamase enzyme production^22^ ^,^ ^23^.

The phenotypic analysis of antimicrobial resistance included 11 antimicrobials from 5 classes: 5 beta-lactams (oxacillin, cefoxitin, cefazolin, cefuroxime, and cephalothin), 2 aminoglycosides (amikacin and gentamicin), 1 macrolide (erythromycin), 1 quinolone (ciprofloxacin), 1 sulfonamide (sulfatrim), and 1 lincosamide (clindamycin). Resistance rates were 100 % (42/42) for oxacillin and cefoxitin, 98 % (41/42) for clindamycin and erythromycin, 86 % (36/42) for gentamycin, 81 % (36/42) for cefuroxime, 74 % (31/42) for sulfatrim, 60 % (25/42) for cefazolin, 98 % (41/42) for clindamycin and erythromycin, 57 % (24/42) for amikacin, and 36 % (41/42) for cephalothin. According to Tavares, resistance of CoNS to antimicrobials has been increasing, and oxacillin resistance is often associated with resistance to macrolides, aminoglycosides, tetracyclines, mupirocin, and clotrimazole because of their similar biochemical mechanisms^24^.

All samples showed resistance to 3 or more classes of antimicrobials, and 16 samples showed resistance to at least 10 of the 11 antimicrobials tested. This high resistance rate corroborates the literature regarding the high capacity of CoNS to develop antimicrobial resistance^17^ ^,^ ^18^.

These results show that the equipment and surfaces analyzed are CoNS reservoirs and thus should be considered effective transmission and dissemination means of HAIs in the analyzed ICU. Our work was limited to investigating only the role of some ICU surfaces considered potential sources for transmission and dissemination of HAIs. It could be considered a study limitation once CDC guidelines for environmental infection control^25^ suggest other aspects that should be also investigated as key points related to HAI incidence. Knowledge of airborne sampling techniques to monitor the effectiveness of air, ventilation, and water systems performance, and other methods for the management of HAIs are also recommended by the CDC guidelines^25^.

We also considered the limitation of this study regarding surface microorganism sampling planning. However, the results reflected the presence of multidrug-resistant microorganisms on the analyzed hospital surfaces. A comprehensive study with a larger number of samples, including collections at other points (patients’ and healthcare professionals’ hands), also including air sampling and co-relating with patient data, could better elucidate the profile of the bioburden found in the hospital.

Despite these limitations, the results should be a warning for healthcare professionals, hygiene service companies, and healthcare institution managers regarding the importance of correct hand hygiene and adequate cleaning and decontamination of the environment and equipment, as the isolation of CoNS suggests failures in the process of surface and equipment cleaning and disinfection performed daily in ICUs. Furthermore, the failure in the cleaning and disinfection process indicates that the sampling sites could eventually be reservoirs for several microorganisms that could have an impact on public healthcare. This makes it even more important for hospitals to strive for excellence when it comes to infection control in order to protect workers and patients as well as their family members. Hand hygiene combined with the correct decontamination procedure of surfaces and equipment is fundamental to control the spread of microorganisms and HAIs.
