# Erratum

**DOI:** 10.1590/0037-8682-0244-2020b

**Published:** 2020-12-18

**Revista da Sociedade Brasileira de Medicina Tropical/*Journal of the Brazilian Society of Tropical Medicine***

**Title:** Identification and characterization of methicillin-resistant *Staphylococcus* spp. isolated from surfaces near patients in an intensive care unit of a hospital in southeastern Brazil 

**Vol.:53:(e20200244): 2020** - doi: https://doi.org/10.1590/0037-8682-0244-2020 - AUTHOR

**Page 1/1**

**Anna Christina de Ameida**

Should read:

**Anna Christina de Almeida**

